# Involvement of serotonin and eicosanoids in the rat paw oedema response to the essential oil of Pilocarpus spicatus

**DOI:** 10.1155/S0962935192000255

**Published:** 1992

**Authors:** J. C. R. Silva, V. S. N. Rao

**Affiliations:** 1 Departamento de Fisiologia e Farmacologia Centro de Ciências da Saúde Universidade Federal do Cearfi. Caixa Postal–657. Fortaleza CE 60.O00 Brazil

## Abstract

Subplantar injection of Pilocarpus spicatus essential oil (PSEO), induced rat hindpaw oedema in a dose-dependent manner. The time course study revealed that when compared to carrageenan-induced oedema, the oedema response to PSEO was greater at 1 h post-injection, and thereafter remained relatively constant until 5 h post-injection. By 24 h, it was still at almost the 50% level. This effect of PSEO was characterized using several inhibitors of oedema formation. Pretreatment with the H_1_-receptor antagonist chlorpheniramine did not affect this response, while a significant reduction of paw oedema was achieved with the serotonin antagonist methysergide, but only 1 h and 2 h after injection of PSEO. The oedemagenic activity of PSEO was also suppressed by pretreating the rats with the eicosanoid synthesis inhibitors, phenylbutazone, EP 10161 and dexamethasone. This last drug showed the greatest potency. These findings suggested a probable injury to dermal mast cells and liberation of arachidonate metabolites and eicosanoids at the late phase of oedema induced by PSEO.


**Research Paper**

Mediators of Inflammation, 1, 167–169 (1992)

### Involvement of serotonin and eicosanoids in the rat paw oedema response to the essential oil of *Pilocarpus spicatus*

J. C. R. Silva and V. S. N. Rao[CA]

Departamento de Fisiologia e Farmacologia, Centro de Ciências da Saúde, Universidade Federal do Ceará. Caixa Postal–657. 60.000–Fortaleza–CE, Brazil

[CA] Corresponding Author

**SUBPLANTAR injection of *Pilocarpus spicatus* essential oil (PSEO), induced rat hindpaw oedema in a dose-dependent manner. The time course study revealed that when compared to carrageenan-induced oedema, the oedema response to PSEO was greater at 1 h post-injection, and thereafter remained relatively constant until 5 h post-injection. By 24 h, it was still at almost the 50% level. This effect of PSEO was characterized using several inhibitors of oedema formation. Pretreatment with the $H_1$-receptor antagonist chlorpheniramine did not affect this response, while a significant reduction of paw oedema was achieved with the serotonin antagonist methysergide, but only 1 h and 2 h after injection of PSEO. The oedemagenic activity of PSEO was also suppressed by pretreating the rats with the eicosanoid synthesis inhibitors, phenylbutazone, EP 10161 and dexamethasone. This last drug showed the greatest potency. These findings suggested a probable injury to dermal mast cells and liberation of arachidonate metabolites and eicosanoids at the late phase of oedema induced by PSEO.**

**Key words:** Eicosanoids, essential oil, *Pilocarpus spicatus*, rat hindpaw oedema, serotonin.

### Introduction

Experimental acute inflammation can be produced by subplantar injection of a number of irritant substances in the hindpaw of rat. Among those of special interest is carrageenan, a sulphated saccharide derived from the red seaweed (*Bucheuma spinosum*) that induces hindpaw oedema. Carrageenan-induced oedema involves predominantly the mediation of cyclooxygenase products of acid arachidonate[1] and this oedema is suppressed by all nonsteroidal anti-inflammatory drugs (NSAIDS).[2] Although this model system bears little relation to human disease conditions, it has significant predictive value for clinically useful anti-inflammatory agents.[3–5] However, there is controversy about the effects of these drugs on the response to carrageenan. For example, compounds of gold, the most efficacious drugs for rheumatoid arthritis, have absolutely no effect on carrageenan-induced oedema.[6] Therefore, the search for novel model systems continues in the development of new anti-inflammatory drugs.

The present paper describes the oedemagenic potential of *Pilocarpus spicatus* essential oil (PSEO) in the hindpaw of rat and the mediator substances involved in its action.

### Materials and Methods

*Animals:* Male Wistar rats (120–180 g), housed at 25–27°C and maintained on standard pellet diet (Purina Chow) and water *ad libitum*, were randomly assigned to various treatment groups. Six animals were included in each group.

*Drugs and chemicals:* The essential oil of *P. spicatus* was supplied by the Natural Products Laboratory of the Federal University of Ceará, Fortaleza, Brazil and lambda carrageenan was purchased from Sigma Chemical Company, St Louis, USA. The other drugs were chlorpheniramine (Schering, São Paulo, Brazil), dexamethasone (Upjohn, São Paulo, Brazil), EP 10161 (Institut Henri Beaufour, Le Plessis, France), phenylbutazone (Geigy, São Paulo, Brazil) and methysergide (Sandoz, São Paulo, Brazil). PSEO was emulsified in Tween 80 (9:1 w/w) and diluted with 154 mM saline. EP 10161 was suspended in 5% DMSO and all other drugs were dissolved in 154 mM saline.

*Paw oedema assay:* Using unanaesthetized rats, PSEO (2.5% and 5%) or carrageenan (1%) was injected into the subplantar surface of the right hindpaw in a volume of 0.1 ml/rat. An equal volume of vehicle was injected into the contralateral paw. The volumes of both hindpaws up to the ankle joint were measured with a plethysmometer prior to and at different periods (1 h, 2 h, 3 h, 4 h, 5 h and 24 h) after induction of oedema. The difference in the volume between the hindpaws was a measure of the oedema present (ml).

*Inhibition of oedema by drugs:* Unanaesthetized rats that had been fasted overnight were treated with a single

© 1992 Rapid Communications of Oxford Ltd dose of chlorpheniramine (10 mg/kg, i.p.), methysergide (1 mg/kg, p.o.), phenylbutazone (100 mg/kg, p.o.), EP 10161 (20 mg/kg, i.p.), dexamethasone (0.5 mg/kg, i.p.) or 154 mM saline (2 ml/kg, i.p.). One hour after drug administration, animals were given 0.1 ml of 5% PSEO into the subplantar region of the right hindpaw. An equal volume of vehicle was injected into the contralateral paw. The paw volume measurements were carried out as described earlier by a plethysmometer at different periods following PSEO injection. The mean paw oedema values for each drug treatment were established and compared with vehicle-treated control. The data were evaluated by use of ANOVA and *t*-independent test. Statistical significance was assigned for $p < 0.05$.

### Results

The effect of graded doses of PSEO (2.5 and 5 mg) and carrageenan (1 mg) on the magnitude of hindpaw oedema is shown in Figure 1. Subplantar injection of PSEO induced a dose-related elevation of paw oedema at all time points. The oedemagenic effect of 5 mg PSEO was greater than that of carrageenan at 1 h post-injection, attained the same peak level at 3 h, remained relatively constant up to 5 h and was still pronounced (almost 50% of peak level) at 24 h.

Figure 2 shows the ability of some of the test drugs to inhibit the oedema induced by 5 mg of PSEO. The PSEO-induced rat hindpaw oedema was not affected by chlorpheniramine, while paw swelling that occurred at 1 h and 2 h was significantly reduced by methysergide. Methysergide was not effective at all other time points. The oedema caused by PSEO was also suppressed by phenylbutazone, EP 10161 and dexamathasone. At the peak effect of PSEO, the extent of oedema inhibition was 25%, 41% and 65% for phenylbutazone, EP 10161 and dexamethasone, respectively.

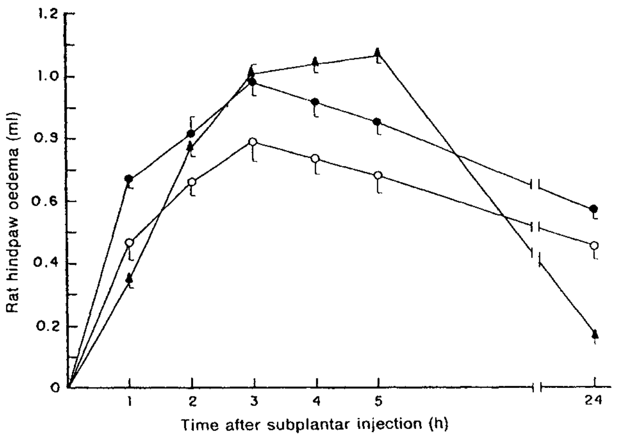

FIG. 1. Time course of oedema after subplantar injection of PSEO and carrageenan in rat. PSEO, 2.5 mg (○); PSEO, 5.0 mg (●); and carrageenan, 1 mg (▲). Means ± SE of data from six animals.

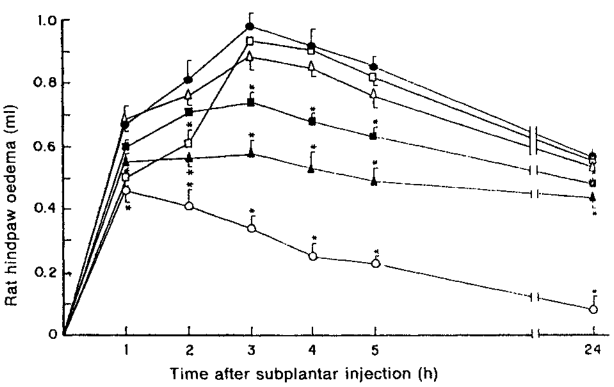

FIG. 2. Effects of drug pretreatment on the time course of rat hindpaw oedema induced by subplantar injection of 5 mg PSEO. Vehicle control (●): chlorpheniramine, 10 mg/kg, i.p. (△); methysergide, 1 mg/kg, p.o. (□): phenylbutazone, 100 mg/kg, p.o. (■): EP 10161, 20 mg/kg, i.p. (▲); and dexamethasone, 1.5 mg/kg, i.p. (○). Drugs were administered 1 h before the subplantar injection of PSEO. Means ± SE of data from six animals. $^*p < 0.05$.

At 24 h, dexamethasone almost abolished the oedema (98% inhibition), while EP 10161 showed 23% inhibition. Phenylbutazone had no significant effect at this time.

### Discussion

The results of the present study show that PSEO is highly potent in promoting a local oedematous reaction. This reaction is dose-dependent and has a time course of action very similar to the one we obtained with carrageenan. The action of PSEO, however, seems more potent because it demonstrated a greater magnitude of oedema than carrageenan at 1 h and 2 h post-injection.

In order to characterize the oedema response to PSEO, a number of specific pharmacological antagonists were utilized. Pretreatment with the $H_1$-receptor antagonist chlorpheniramine was without any effect on PSEO response, while methysergide, a serotonin antagonist significantly inhibited the response at an earlier time. This observation suggested a possible injury to dermal mast cells and release of serotonin in the early phase of oedematous reaction. In agreement with the results are the reports by others that serotonin but not histamine is largely responsible for hindpaw oedema induced by compound 48/80 and carrageenan.[1,7] Cyclooxygenase inhibition does not seem to affect serotonin-induced paw oedema in rats[8] and probably for this reason phenylbutazone did not show any influence on early phase oedema reaction to PSEO. Nevertheless, the finding that phenylbutazone, EP 10161 and dexamethasone (cyclooxygenase, lypoxygenase and phospholipase $A_2$ inhibitors, respectively) were potent in suppressing late phase oedema response to PSEO established that eicosanoids are the major inflammatory mediators. The rank order potency of these inhibitor drugs is dexamethasone > EP 10161 > phenylbutazone. The fact that EP 10161 produces a greater inhibitory effect than phenylbutazone indicates that leukotrienes are more important mediators than prostaglandins in this model. In contrast, carrageenan-induced oedema involves predominantly the mediation of prostaglandins.[1]

In the present study, dexamethasone produced a more potent block in the oedemagenic effect of PSEO. Dexamethasone has been reported to inhibit increased vascular permeability independent from inhibition of phospholipase $A_2$.[9] It is possible that similar mechanisms might underlie the inhibitory action of dexamethasone against PSEO-induced inflammatory response.

The two major constituents of PSEO were methylnonylketone ($\sim$80%) and methylundecylketone ($\sim$10%). The oil also contains some minor constituents such as terpenes and phenols. It remains to be verified whether one or more of these constituents are responsible for the induction of PSEO-induced inflammatory reaction. It is probable that PSEO, besides promoting mast cells degranulation, may activate phospholipase $A_2$ enzyme in vascular endothelial cells and may release arachidonic acid from membrane phospholipids. Arachidonate metabolism through cyclooxygenase and lipoxygenase pathways is known to produce highly potent inflammatory mediators, namely prostaglandins, thromboxanes and leukotrienes.[10–12] Therefore it is reasonable to assume that PSEO-induced hindpaw oedema in rat involves serotonin at the early phase and eicosanoids, particularly leukotrienes, at the late phase.

### References

1. Vinegar R, Truax JF, Jeffrey L, *et al.* Pathway to carrageenan-induced inflammation in the hind limb of the rat. *Fed Proc* 1987; **46**: 118–126.
2. Vane JR. Inhibition of prostaglandin synthesis as a mechanism of action for aspirin like drugs. *Nature New Biol* 1971; **231**: 232–235.
3. Lombardino JG, Otterness KG, Wiseman EH. Acid anti-inflammatory agents—correlations of some physical, pharmacological and clinical data. *Arzmeimittel-Forsch* 1985; **25**: 1629–1635.
4. Van Arman CG. Anti-inflammatory drugs. In: Vane JR, Ferreira SH, eds. *Handbook of Experimental Pharmacology*. Berlin: Springer Verlag, 1979; 75.
5. Otterness IG, Gans DG. Nonsteroidal anti-inflammatory drugs: an analysis of the relationship between laboratory animal and clinical doses, including species scaling. *J Pharm Sci* 1988; **77**: 790–795.
6. Goodwin JS. Mechanisms of action of nonsteroidal anti-inflammatory agents. *Amer J Med* 1984; **77**: 57–64.
7. Parrat JR, West GB. Inhibition by various substances of oedema formation in the hind-paw of the rat induced by 5-hydroxytryptamine, histamine, dextran, eggwhite and compound 48/80. *Br J Pharmac Chemother* 1958; **13**: 65–70.
8. Winter CA. Nonsteroidal anti-inflammatory drugs. Garantini S, ed. *Excerpta Medica* 1964: 190–202.
9. Tsurufuji S, Sugio K, Takemase F. The role of glucocorticoid receptor and gene expression in the anti-inflammatory action of dexamethasone. *Nature* 1979; **280**: 408–410.
10. Boot J, Dawson W, Kitchen W. The chemotactic activity of thromboxane $B_2$. A possible role in inflammation. *J Physiol* 1976; **257**: 47.
11. De Clerck F, Loots W, Somers Y, Van Gorp L, Verheyen A, Wouters L. Thromboxane $A_2$-induced vascular endothelial cell damage and respiratory smooth muscle contraction: inhibition by flunarizine, a $Ca^{2+}$-overload blocker. *Arch Int Pharmacodyn Ther* 1985; **274**: 4–23.
12. Zipser RD, Laffi G. Prostaglandins, thromboxanes and leukotrienes in clinical medicine. *West J Med* 1985; **143**: 485.

ACKNOWLEDGEMENTS. This research was supported by the National Research Council-CNPq and FINEP. We wish to thank Prof. M. Andrade Neto, Natural Products Laboratory of the Federal University of Ceará, Fortaleza, Brazil, who kindly supplies the *P. spicatus* essential oil.

**Received 5 February 1992; accepted in revised form 18 March 1992**
